# Conservation implications of turtle declines in Australia’s Murray River system

**DOI:** 10.1038/s41598-019-39096-3

**Published:** 2019-02-13

**Authors:** J. U. Van Dyke, R. –J. Spencer, M. B. Thompson, B. Chessman, K. Howard, A. Georges

**Affiliations:** 1 School of Science and Health, Hawkesbury Institute for the Environment, Western Sydney University, Locked Bag 1797, Penrith, NSW 2751 Australia; 2 School of Environmental Sciences, Institute for Land, Water, and Society, Charles Sturt University, Albury-Wodonga Campus, Albury, NSW 2640 Australia; 3 School of Life and Environmental Sciences, Heydon-Laurence Building (A08), University of Sydney, Sydney, NSW 2006 Australia; 4 Centre for Ecosystem Science, University of New South Wales, Sydney, NSW 2052 Australia; 5 Institute for Applied Ecology, University of Canberra, Canberra, ACT 2601 Australia

## Abstract

Conservation requires rapid action to be effective, which is often difficult because of funding limitations, political constraints, and limited data. Turtles are among the world’s most endangered vertebrate taxa, with almost half of 356 species threatened with extinction. In Australia’s Murray River, nest predation by invasive foxes (*Vulpes vulpes*) was predicted to drive turtle declines in the 1980s. We assessed populations of the broad-shelled turtle (*Chelodina expansa*), eastern long-necked turtle (*C. longicollis*), and Murray River turtle (*Emydura macquarii*) in the Murray River and some of its associated waterways. Our results suggest that the predicted decline is occurring. All three species are rare in the lower Murray River region, and were undetected in many locations in South Australia. Moreover, *E. macquarii* had considerable population aging almost everywhere, possibly due to comprehensive nest destruction by foxes. *Chelodina longicollis* also had population aging at some sites. Sustained low recruitment has potential to lead to collapses as turtles age, which is particularly worrying because it was predicted over 30 years ago and may have already occurred in South Australia. Our results show that turtle declines were not mitigated since that prediction. If the crash continues, a vertebrate guild responsible for considerable nutrient cycling in the aquatic ecosystem will disappear. Our results highlight a worst-case outcome when species declines are predicted, but insufficiently mitigated.

## Introduction

Earth’s sixth mass extinction is currently under way^1–4^. Although a number of mechanisms exist for predicting species’ extinction risks due to varied threats^5,6^, the complex synergies that drive extinction severely hamper our ability to protect declining species^7^. However, conservation success depends on rapid action^8^. Here, we describe the ongoing decline of a freshwater turtle community. The decline was first predicted over 30 years ago^9^, and we show that subsequent mitigation has been inadequate to prevent it.

Worldwide, about half of the 356 extant turtle species are listed as vulnerable or worse by conservation agencies^10^. Adult turtles are overharvested for both human consumption and the pet industry^11,12^, can experience high road mortality^13,14^, and are killed by human-introduced predators when they nest or disperse^10,15^. Turtles are struck by boats^16^, drowned in fishing nets and other human structures^17,18^, and wounded or killed by anglers^19,20^. Mortality of eggs and young has also increased, primarily because of poaching and predation by introduced predators^9,21,22^. Humans introduce turtles into novel ecosystems, which can lead to hybridization and genetic introgression^23,24^, and competition with native species^25^. Finally, novel diseases have potential to cause rapid extinctions, as nearly occurred in the Bellinger River snapping turtle (*Myuchelys georgesi*) in 2015^26–28^, and may be occurring in other species^29,30^.

In addition to acute threats, turtles are susceptible to environmental degradation. Increasing global temperatures may drive changes in population sex ratios in species with temperature-dependent sex determination^31^. Drought may cause both reduced breeding success and outright population declines in freshwater turtles in both North America and Australia^32,33^. These threats may be exacerbated by water flow regulation and extraction^34^. Increased salinization of freshwater habitats during droughts is especially dangerous for some species^35,36^. As long-lived species, often with catholic diets, turtles bioaccumulate high contaminant loads in polluted systems^37,38^, potentially with reproductive consequences^39,40^. Urbanization and habitat fragmentation increase turtle mortality^41,42^. Furthermore, even when threats to turtles can be mitigated, recovery is often slow^43^, due to long generation times and high rates of attrition at egg and juvenile life-stages^44^. Indeed, a recent study found no recovery in a *Chelydra serpentina* population that suffered mass mortality in the 1980 s^45^.

In Australia, about half of the 25 extant species of freshwater turtle are currently listed as vulnerable, endangered, or critically endangered. Invasive predators have been highlighted as a primary threat. More than 30 years ago, exceptionally high rates of nest predation by invasive red foxes (*Vulpes vulpes*) were predicted to drive freshwater turtle declines in the Murray River^9^. Foxes destroyed such large numbers of turtle nests (>93% per year) that juvenile recruitment was reduced to nearly zero, and only adult turtles remained abundant within the population^9^. Management of invasive predators has thus been one of the primary mechanisms for preventing turtle declines in Australia^15,22,44,46^. However, its effectiveness has not been comprehensively assessed. Only one study^33^ has compared turtle populations in the Murray River (*Chelodina expansa*, *Chelodina longicollis*, and *Emydura macquarii*) at multiple timepoints. Its results are alarming, as abundances of *C. longicollis* and *E. macquarii* declined by 69–91% between 1976 and 2011, and juvenile proportions of turtle populations plummeted^33^. However, this study was conducted at only at a few sites in south-central New South Wales and it is unclear whether the declines are consistent along the entire Murray River catchment.

Here, we report a rapid assessment of turtle populations throughout the southern Murray River catchment. Our aim was twofold: (1) to establish a baseline understanding of current turtle population status throughout the southern Murray River catchment, including identifying likely “hotspots” of turtle declines for future study and management actions; and (2) to identify geographic trends in turtle population status that could lead to focused hypothesis tests on the causes of any identified turtle declines. We assessed populations of all three species native to the river system, including the broad-shelled turtle (*C. expansa*), eastern long-necked turtle (*C. longicollis*), and Murray River turtle (*E. macquarii*).

We used a dual approach to test for differences in population status across the Murray River catchment^33^. First, we used catch-per-unit-effort (CPUE) as an index of relative abundance of each turtle species. We assume that sites with a low CPUE are sites where turtle densities are lower, whereas sites with high CPUE are sites where turtle densities are higher. Second, we tested for population differences in the proportions of juvenile and large adult turtles. Here, we used size as an index of age, such that large adults should be relatively old, and juveniles should be young. We followed Chessman^33,47^ in setting the maximum sizes (straight carapace length; SCL) for juvenile turtles: 220 mm for *C. expansa*, 180 mm for *C. longicollis*, and 190 mm for *E. macquarii*. We arbitrarily considered turtles with SCLs larger than 350 mm for *C. expansa*, 230 mm for *C. longicollis*, and 250 mm for *E. macquarii* to be “large” adults. Chessman^33^ and Thompson^9,48^ argued that the proportion of juveniles was an indicator of recent recruitment, whereas the proportion of large adults could indicate population aging. Thus, populations dominated by large adults likely experience low recruitment, and are potentially at risk of collapse when those turtles die of old age. In contrast, populations with large numbers of juveniles experience relatively high levels of recruitment and may have a lower risk of collapse.

## Results

Throughout the study, we caught 36 *C. expansa*, 251 *C*. *longicollis*, and 815 *E. macquarii*. Recapture rates were low, and we recaptured only two *C. expansa*, eight *C. longicollis*, and 18 *E. macquarii*. However, we re-captured one *E. macquarii* four times at Lake Mulwala. In South Australia, we captured 8 *E. macquarii* that appeared to be marked from previous studies, but never re-captured these subsequently.

**Figure 1:**
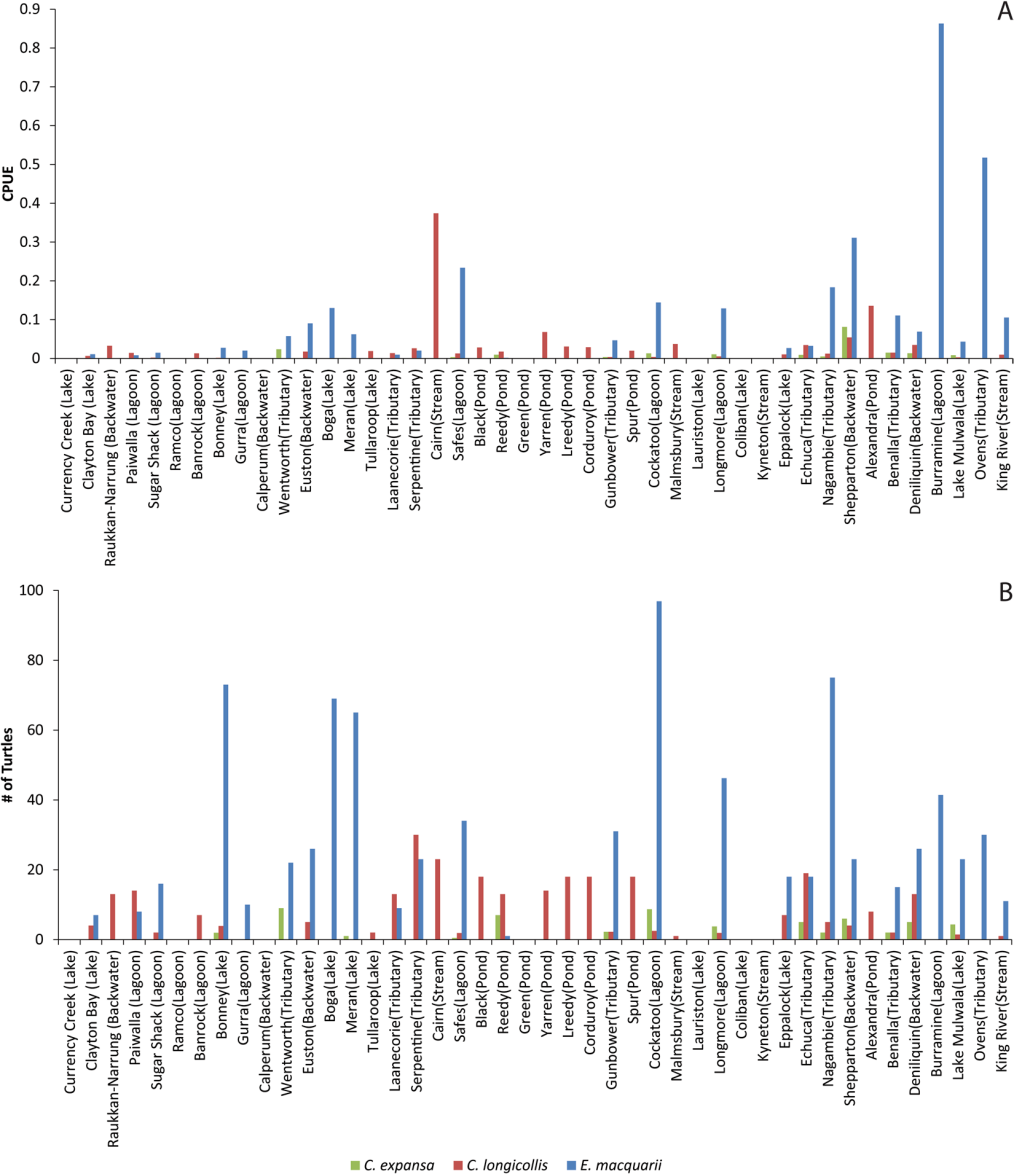
(**A**) Turtle CPUE (turtles per trap-hour) and (**B**). Total number of each species caught for each site trapped in the study, with site names and designations. Sites are ordered left-right from downstream-upstream. Far upstream sites where 0 turtles were caught and which are likely beyond extant turtle ranges have been removed for clarity.

**Figure 2:**
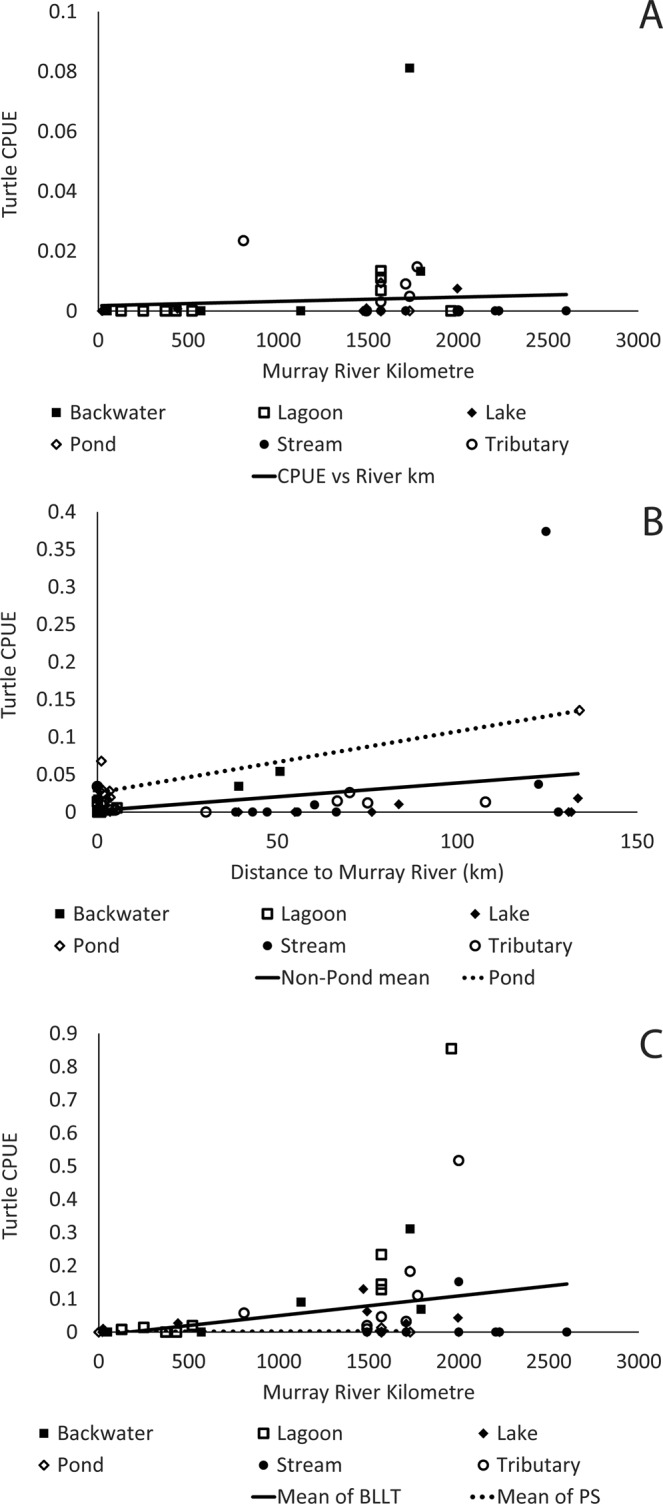
Mean catch-per-unit-effort (CPUE; turtles per trap-hour) of (**A**) *Chelodina expansa* increased slightly with increasing distance from the Murray River mouth, regardless of wetland type, but this relationship was not retained in reduced models (Table S1). (**B**) CPUE of *C. longicollis* increased with increasing distance to the Murray River, and was higher in ponds than in other types of wetlands. The relationship with distance from the Murray River was not retained in a reduced model, but the difference between ponds and other wetlands was (Table S1). (**C**) CPUE of *Emydura macquarii* increased with distance from the Murray River mouth and also significantly higher in backwaters, lagoons, lakes and tributaries (BLLT) than in ponds and streams (PS). Lines on each graph represent best-fit regressions identified in log-linear ANCOVAs (Table 1).

CPUE and numbers of turtles caught varied across the southern Murray River catchment within each species (Table 1, Fig. 1). Because we accounted for trapping effort in each of the following analyses, we focus only on CPUE in our results, from here forward. Despite species-specific statistical patterns in our results, CPUEs of all three species were generally highest in the mid-Murray region (River Distance ~1500–2000 km), which corresponds to north-central Victoria and south-central New South Wales (Fig. 1). In *C. expansa*, CPUE was low across all sites (Fig. 1), but significantly increased with increasing distance from the river mouth (Fig. 2A; Table 1). CPUE of *C. expansa* did not differ with any other factor tested (Table 1). The relationship between CPUE and river kilometre was not retained when we removed sites with low or high trap effort (Table S1).

**Table 1:** Statistical results of log-linear ANCOVA analyses on the CPUE of each turtle species in the southern Murray River catchment.

Species	Effect	F	Num df	Den df	P
*C. expansa*	Distance to Murray River	0.10	1	40	0.751
	Distance to Permanent	1.86	1	40	0.180
	Mean Precipitation	0.01	1	40	0.911
	Mean Temperature	2.04	1	40	0.161
	**River Kilometre**	**6.56**	**1**	**40**	**0.014**
	Trapping Effort	0.01	1	40	0.955
	Wetland Type	0.73	5	40	0.608
*C. longicollis*	**Distance to Murray River**	**5.80**	**1**	**40**	**0.021**
	Distance to Permanent	2.29	1	40	0.138
	Mean Precipitation	1.80	1	40	0.192
	Mean Temperature	0.60	1	40	0.443
	River Kilometre	0.79	1	40	0.378
	Trapping Effort	0.06	1	40	0.808
	**Wetland Type**	**5.61**	**5**	**40**	**<0.001**
*E. macquarii*	Distance to Murray River	0.06	1	40	0.810
	Distance to Permanent	0.01	1	40	0.977
	Mean Precipitation	0.09	1	40	0.766
	Mean Temperature	3.41	1	40	0.103
	**River Kilometre**	**9.07**	**1**	**40**	**0.005**
	Trapping Effort	0.68	1	40	0.415
	**Wetland Type**	**5.42**	**5**	**40**	**<0.001**

Factors that were statistically significant in each model are in bold font.

*Chelodina longicollis* CPUE significantly increased with increasing distance from the Murray River, and these relationship varied across wetland types (Fig. 2B; Table 1). Specifically, *C. longicollis* CPUE was higher in pond habitats than in all other types of habitats (Tukey P < 0.05; Fig. 2B). After removal of low-trap-effort sites the relationships between CPUE and distance from the Murray River were no longer significant (Table S1). However, there was still a significant difference in CPUE among wetland types (Table S1), and ponds had higher mean CPUE than did lakes, streams, and lagoons (Tukey P < 0.05), but not tributaries or backwaters (Tukey P > 0.05).

*Emydura macquarii* CPUE was generally higher than for the other two species (Fig. 1), and varied across wetland types and with river distance (Table 1). Specifically, CPUE increased with increasing distance from the river mouth (Fig. 2C). After accounting for river distance effects, mean *E. macquarii* CPUE was lower in ponds and streams than in all other habitats (Fig. 2C; Tukey *P* < 0.05). Unlike the CPUE results for the other turtle species, the statistical results for *E. macquarii* were not affected by the removal of sites with low or high trap effort (Table S1).

Proportions of juvenile and large adult turtles varied among wetland types and species. We caught *C. expansa* in sufficient numbers (at least five) to be included in our analysis at only five locations (Fig. 1), so we did not analyze *C. expansa* size structures. Two of these sites (tributaries at Echuca and Wentworth) had no obvious bias to juveniles or large adults. Two sites (Reedy and Cockatoo lagoons) had juvenile proportions of 22.2% and 50.0%, respectively. Reedy Lagoon also had 11.11% large adult females, and a backwater at Shepparton had 16.7% large adult females.

**Figure 3:**
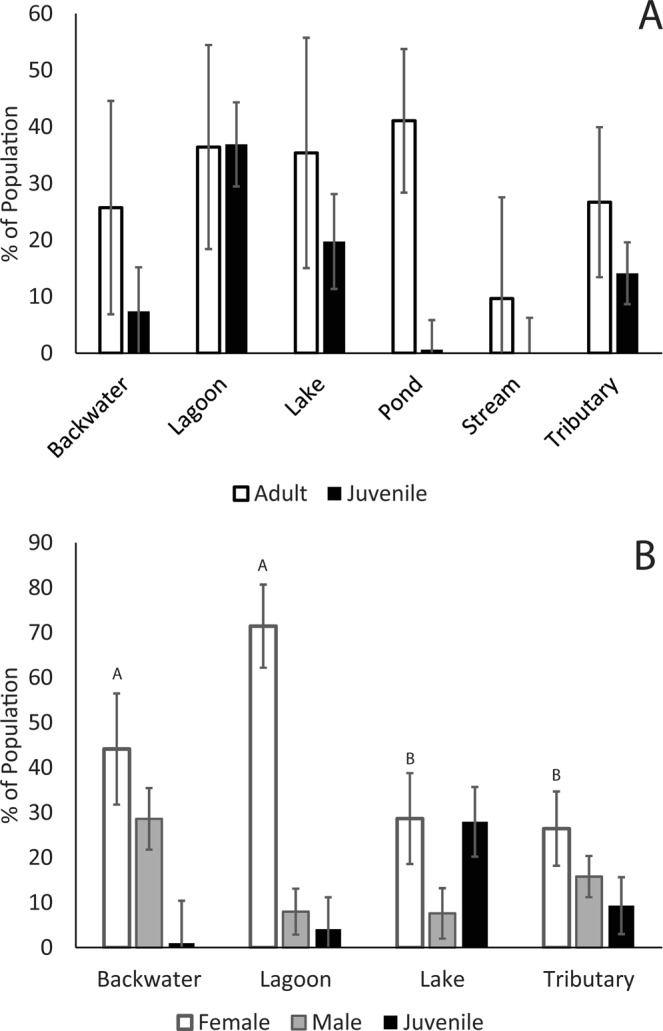
Mean percentages of populations of (**A**) *Chelodina longicollis* made up by large adults and juveniles, and (**B**) *Emydura macquarii* large females, large males, and juveniles. We present the data for both species as percentages for clarity, but we accounted for the number of turtles caught in each site using a log-linear ANCOVA on the raw numbers of each sex-size group, rather than on percentages. Error bars represent ± 1 SE, and letters indicate pairwise significant differences between size/sex number within each habitat type. Mean percentages do not sum to 100% because we did not include the percentages of intermediate-aged turtles in these analyses.

Numbers of juvenile and large adult *C. longicollis* differed uniquely across wetland types, and both increased as the total number of turtles caught increased (Table 2; Fig. 3A). We caught significantly more large adult*s* than juveniles in pond wetlands (Tukey P < 0.05), but there were no significant differences within any other wetland type (Fig. 3A). Juveniles were also rare in backwaters and streams, but the numbers of large adults in these wetland types varied widely across individual sites (Fig. S2). No other factors were significant (Table 2).

**Table 2:** Statistical results of log-linear ANCOVA analyses on the age structures of *C. longicollis* and *E*.

Species	Effect	F	Num df	Den df	P
*C. longicollis*	Distance to Murray River	1.02	1	11	0.334
	Distance to Permanent	2.99	1	11	0.112
	Mean Precipitation	1.55	1	11	0.239
	Mean Temperature	0.4	1	11	0.540
	**Number of Turtles**	**10.44**	**1**	**11**	**0.008**
	River Kilometre	0.24	1	11	0.633
	Sex	2.96	1	11	0.113
	Wetland Type	2.3	5	11	0.116
	Sex*Wetland Type	**4.04**	**5**	**11**	**0.025**
*E. macquarii*	Distance to Murray River	0.18	1	39	0.670
	Distance to Permanent	1.29	1	39	0.263
	Mean Precipitation	1.68	1	39	0.202
	Mean Temperature	0.09	1	39	0.768
	**Number of Turtles**	**5.71**	**1**	**39**	**0.022**
	River Kilometre	0.08	1	39	0.774
	**Sex**	**4.88**	**2**	**39**	**0.013**
	Wetland Type	0.36	4	39	0.835
	Sex*Wetland Type	**2.81**	**8**	**39**	**0.015**

*Macquarii* in the southern Murray River catchment. Factors that were statistically significant in each model are in bold font. “Sex” represents a composite of both sex and age class.

In *E. macquarii*, numbers of juvenile and large adult turtles also varied uniquely among wetland types, and both increased with the total number of turtles caught (Table 2). We caught significantly more large females than juveniles within both backwaters and lagoons (Tukey P < 0.05; Fig. 3B). We also caught more large females than large males within lagoons (Tukey P < 0.05; Fig. 3B). There were no within-habitat differences in the numbers of large male, large female, or juvenile turtles in lakes or tributaries (Fig. 3B), and no other factors were significant. Large females were common at almost all sites we trapped (Fig. S3), but backwaters and lagoons had very high proportions of large adult females (Fig. 3B). Juveniles were rare across all sites, but were relatively common in lakes (Fig. 3B). Large males were consistently distributed throughout all wetland types (Fig. 3B). Notably, locations and wetland types with high proportions of large females, as a consequence, had low proportions of males of all sizes, which may indicate a sex-ratio bias towards females, especially in backwaters and lagoons. We did not catch enough *E. macquarii* in stream or pond wetland types to include them in this analysis.

## Discussion

Australia is now at the stage where the effects of invasive species and habitat destruction are being observed through species declines and extinction events. Across Australia, nearly half of all turtle species are listed as vulnerable or worse by state or federal agencies^49^. Turtle declines of up to 91% have been observed in sections of the Murray River^33^. A recent novel disease has also caused the near-extinction of an Australian freshwater turtle (*Myuchelys georgesi*), and may be a symptom of deteriorating water quality and climate change^28^. Here, our results provide further evidence that freshwater turtle populations in southern Australia are declining.

Catch-per-unit-effort data indicate that overall turtle abundance decreases as the Murray River approaches the ocean. Notably, we caught zero or very few turtles of any species at several locations in South Australia, including Calperum Station, Ramco Lagoon, and Currency Creek. Turtle numbers (relative to trapping effort) were higher in the mid- and upper-Murray regions. However, 10 of the sites where we caught zero turtles of any species were far upstream on the Murray River or its tributaries, and were much colder and faster flowing than other sites. These locations may not be suitable for turtles due to their ectothermic physiology. Even at the sites nearby where we did catch turtles, CPUE of *E. macquarii* was always low, possibly because our trapping exceeded their current range and extended beyond their preferred habitats^50^. By contrast, *C. longicollis* was occasionally very abundant in upstream, rocky stream sites, so it is unclear whether we extended beyond their preferred range or whether they are present but only at low densities at the sites where they were not captured.

*Emydura macquarii* are relatively abundant in some habitats in the mid-Murray region, but their numbers (relative to trapping effort) drop precipitously further downstream. In 8 of 10 sites in South Australia, we caught fewer than 10 *E. macquarii*, despite trapping efforts that were consistent with, or even exceeded, those further upstream. All of these downstream sites were near to other locations where we caught turtles, are well within the reported range of *E. macquarii*, and many probably supported large turtle populations in the past^48^. Across our study, *E. macquarii* was most abundant in backwater, lagoon, lake, and tributary habitats connected or close to the main channels. The habitat associations we detected largely follow the published preferences of the species^50,51^.

*Chelodina longicollis* exhibited higher mean CPUE in ponds disconnected from the main channel than in other sites. In addition, CPUE increased with increasing distance from the Murray River, but this relationship was not robust and disappeared when we removed sites with very low and high trapping effort from our statistical model. Low CPUE close to the river may indicate that the species has declined close to the river^33,44^. However, our results are also consistent with the species’ reported ecology. *Chelodina longicollis* has a broad distribution across the region and is not as tied to permanent water as are other species of turtles^50^. It prefers temporary wetlands in much of the Murray catchment, and is also found in both temporary and permanent wetlands far from the larger rivers of the region^50^. It is capable of migrating long distances overland to access/emigrate from ponds as they fill or dry^52,53^. Thus, *C. longicollis* may still be present in relatively high numbers farther from the river. Whether the differences in CPUE that we detected are due to its habitat preferences or localized declines is unclear, but a long-term comparison does support the hypothesis that a decline may be occurring in the mid-Murray^33^.

*Chelodina expansa* CPUE was low across the entire catchment, and increased with increasing distance from the Murray mouth. The latter trend was not statistically robust, but we caught only one individual in all trapping downstream of Mildura, VIC. *Chelodina expansa* was recently present in this region^34,36^, so our data may reflect our comparatively low sampling effort. We also focused primarily on wetlands adjacent to the river, and did not trap in the river itself, where *C. expansa* may be more abundant^34,36^. We caution that *C. expansa*’s historically low density also means that even proportionally large declines could be difficult to detect. Thus, although *C. expansa* may not have declined in the mid-Murray, it may have always been present in relatively low numbers. Sites where we caught the highest numbers of *C. expansa* again reflect previously-published habitat preferences^50,51^, including backwaters and lagoons with direct connections to the main channel of the Murray River.

The results of our analyses on the proportions of juveniles and large adults within turtle populations are striking, particularly for *E. macquarii*. Even in the habitat types where *E. macquarii* is most abundant, large adult females (>250 mm SCL), on average, account for the largest proportion of the population. Thus, most *E. macquarii* populations are strongly biased to large adult females. In contrast, juveniles made up very small proportions of most populations, which suggests that recruitment rates have been very low in recent years. Our observation of low juvenile numbers is similar to those reported in other demographic studies of *E. macquarii*^9,33^, and highlights that many populations of *E. macquarii* are aging. The trend is possibly a result of ongoing, consistently high rates of nest predation by foxes, which may nearly eliminate juvenile recruitment^15,22,54^. Population aging may also be caused by other factors that reduce recruitment but have not yet been investigated in turtles. The southern Murray catchment is heavily impacted by altered flow regimes^55^ and invasive carp^56^. Resulting changes water turbidity and macrophyte abundance have been linked to changes in *E. macquarii* diet^57^, and we suspect that the loss of macrophytes might also increase predation of hatchling and juvenile turtles by fish and wading birds. Regardless of the cause, our results suggest that ongoing, consistent low recruitment may drive turtle declines in the Murray River, which could lead to a population collapse across the catchment^9,48^.

Several locations are clear exceptions and exhibit relatively high rates of juvenile recruitment. Lake Eppalock on the Campaspe River, Lake Meran, Serpentine Weir on the Loddon River, and Clayton Bay at Lake Alexandrina all exhibited relatively high numbers of juvenile *E. macquarii*. All but Lake Meran also exhibited low numbers of turtles caught, and relatively low CPUE. Lake Eppalock, Lake Meran, and Serpentine Weir experienced complete or near-complete drying during the Millennium Drought^58–61^. Near Clayton Bay, an infestation with the Australian tubeworm (*Ficopomatus enigmaticus*) was reported in the Lower Lakes region in 2008. This emergent condition in turtles is due to high water salinity, which occurred during the Millennium Drought^35^. The worms form calcareous tubes on a range of artificial and natural surfaces and restrict the ability of turtles to swim effectively. Either complete drying or tubeworm encrustation could have killed a number of adult turtles at these sites, which may account for our low CPUE. Interestingly, comparisons of turtle body size against published growth-curves^62^ indicate that the juvenile turtles caught at these sites hatched since the end of the drought in 2010.

Assuming that fox predation on turtle nests is a major driver of the low numbers of juveniles we observed^15,22,54^, it is possible that the Millennium Drought caused such severe crashes in the adult *E. macquarii* populations at these locations that their nests are difficult for foxes to find (e.g. functional response^22^). At very low densities, nests may experience some relief from fox predation as foxes switch to other prey^22^, and at least a limited recovery of the population via juvenile recruitment may be possible, until nest abundance is again high enough for foxes to target. It is also possible that the numbers of turtles we caught are too low to accurately reflect population size structures, particularly at Clayton Bay and Lake Eppalock.

Our age structure analysis for *C. longicollis* did not detect strong geographic or climatic differences. Large adult turtles (>220 mm SCL) were much more common than juveniles within pond habitats, but we detected no other differences. Ponds had relatively large numbers of *C. longicollis*, so it is possible that high densities of turtle nests might attract higher levels of fox predation on eggs at these sites^22^, which would reduce recruitment. Alternatively, some of the ponds included in our sampling completely dry occasionally, and it is possible that hatchling and juvenile turtles are less able to avoid the impacts of wetland drying than are adults, via estivation or migration, though this has not previously been reported^52^. In contrast to *E. macquarii*, we caught relatively high numbers of juvenile *C. longicollis* at some lagoon, lake, and tributary habitats throughout the Murray catchment. In another study, *C. longicollis* juveniles were rare during the Millennium Drought (1996–2010) in the lower Murrumbidgee River, but have been caught with increasing frequency since 2013^51^. Together, these results suggest that *C. longicollis* populations experience unique patterns in juvenile recruitment, and are not as consistently at risk of population aging as are *E. macquarii*. The ability of *C. longicollis* to migrate long distances overland to exploit temporary wetland habitats^52,53^ may allow a greater degree of terrestrial dispersal of turtles of all age groups than occurs in other species, which could buffer against population aging. The same migratory ability may also have allowed adult *C. longicollis* to persist in environments that experienced drying during the Millennium Drought, especially Serpentine Weir.

Together, our data provide evidence for regional declines in freshwater turtles in the Murray River catchment. All three species were detected at low numbers, relative to trapping effort, throughout most of South Australia, but *E. macquarii* populations appear to be at particular risk. Although relatively more abundant at many locations further upstream, *E. macquarii* may be at risk of a collapse even at many of these sites because the majority of *E. macquarii* populations are heavily biased to older females, and juvenile turtles are uncommon. These size structures may be caused by low rates of recruitment. Low recruitment may be due to high rates of fox predation on nests, or to changes to the environments that hatchlings require once they reach the water. Incubating and hatching eggs in captivity to avoid foxes altogether would test the role of foxes in the decline, following Spencer and Thompson^15^. If recruitment improved after release of captive-incubated hatchlings at multiple sites along the Murray River, then foxes might be assumed to be the major cause of turtle losses in recruitment at a landscape scale. Alternatively, if released hatchlings were not recaptured and recruitment remained low even after hatchling release, then other factors, like macrophyte loss, food web disruption, or water quality issues, may be responsible for low recruitment rates. It is also possible that low CPUE is caused by both lack of recruitment and by high adult mortality. The causes of the decline thus require additional study.

Despite turtle declines in South Australia being predicted 30 years ago, that prediction was not followed by extensive study or successful management of freshwater turtles. If further surveys show continued declines in turtles, a major vertebrate guild responsible for considerable nutrient and mineral cycling in the aquatic ecosystem^48,63^, could eventually become extinct. Our results suggest that reassessments of the conservation status and management plans of these species are needed. *Emydura macquarii* is listed as Vulnerable in both South Australia and Victoria, and *Chelodina expansa* is listed as Vulnerable in South Australia and Endangered in Victoria, but none of these species are listed in New South Wales. Furthermore, none are listed at the federal level. By contrast, *C. longicollis* is widespread away from the major waterways of the Murray River system, and further assessments are needed to examine its status away from the catchment, where our study was focused. Finally, it is worth noting that, despite the many threats they face, freshwater turtles are sometimes considered resilient taxa because many species are able to survive in poor quality habitats where other taxa cannot^64–68^.

Long-lived species like turtles are often neglected as at-risk species in need of conservation action, due to the “perception of persistence”^63^. Even in species with relatively high abundance, low rates of recruitment may be catastrophic once the majority of individuals present die of old age^63^. Often it is only when populations crash or a crash is imminent that they become listed for conservation action, yet the life history of long-lived species means that they give us considerable warning. In turtles from the Murray River, this population crash was predicted almost 30 years ago, and our data indicate that it is in progress.

## Methods

### Field Sampling

**Figure 4:**
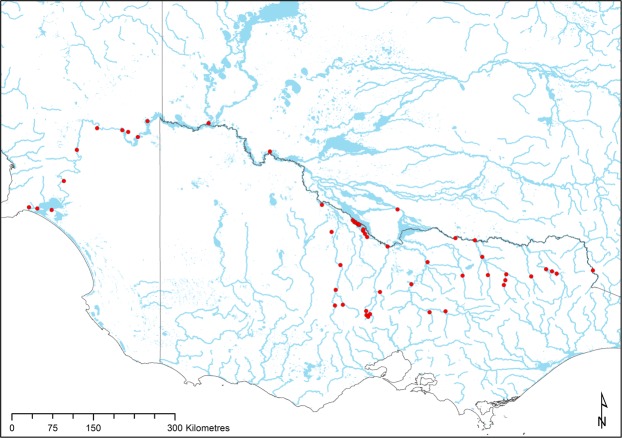
Locations of all trapping sites (dots) in our study area.

We assessed turtle populations at 52 sites throughout the southern Murray River catchment in January-April of 2015–2017 (Fig. 4). January-April in the Murray region coincides with consistently warm and dry weather and high turtle activity^50^. It is after the spring nesting season for *C. longicollis* and *E. macquarii*, but overlaps with the autumn nesting season of *C. expansa*. All upper Murray sites (river km 1730–2600) were assessed in Jan, 2016; mid-Murray sites (river km 1125–1730) in Feb-Mar, 2015 and 2016; and lower Murray sites (river km 19–570) in Jan-early Mar, 2017. Only 2 sites were assessed in early April, 2016: Wentworth and Euston, but maximum air temperatures at these sites exceeded 30 °C and turtles were active. Most, but not all, of our sites were chosen because they were on opposite sides of a dam, weir, or impoundment, to contribute data to a concurrent study on genetic connectivity among *E. macquarii* populations (Berman *et al*. unpubl. data), but were not near the dam or in a weir pool itself. Notably, we did not survey much of the upper Murray (Fig. 4; near Albury, NSW) because of pre-existing data of the concurrent genetic study, which used a different survey method (Berman *et al*. unpubl. data).

We included a range of wetland types in our assessment, and included sites associated with the Murray River itself, tributaries to the Murray River, and anabranches in the Murray River catchment. We defined wetland types by their physical characteristics: backwaters are unrestricted embayments broadly connected to the main channel of a river or lake; lagoons are embayments that are narrowly connected to the main channel of another, larger water body; lakes are sites trapped near the open shoreline of a large natural lake or large reservoir/impoundment; ponds are small wetlands disconnected from any adjacent waterway; streams are small fast-flowing upland waterways with rocky bottoms; and tributaries are large slow-flowing waterways with sand or mud bottoms.

Our trapping schedule evolved over time. To meet the requirements of our concurrent genetic study (Berman *et al*. unpubl. data), we initially aimed to trap at each site until at least 30 *E. macquarii* were caught. In our initial trapping in 2015, we were sometimes able to catch 30 *E. macquarii* using only 3–4 traps at a time overnight, which resulted in high CPUE relative to low trapping effort at Burramine and Ovens (Fig. 1). As our trapping progressed to other locations, our catch rates decreased. Thus, to balance trapping effort with trapping at as many sites as possible, at the majority of sites we used at least 10 traps at a time, set for at least three overnight periods or until we caught at least 25 *E. macquarii*. We switched to 25 minimum *E. macquarii* rather than 30 because in some cases we caught between 25 and 30 *E. macquarii* within 2 nights, and we decided it was more productive to move traps to another site in our survey to ensure we trapped all sites before the active season ended.

There were several exceptions to this rule due to extraneous circumstances. Landowners of Raukkan-Narrung, Banrock Station, Alexandra, Malmsbury, and King River were only able to allow us access for 1-2 nights, and there were limited sites suitable for setting our traps due to high currents and shallow water at Malmsbury and King River. Our sites in Benalla, Tullaroop, and Cairn Curran had anglers present after 1-2 nights, so we removed traps to prevent them from being stolen, which occurred several times early in our study. Finally, we had a side project at Lake Bonney that allowed us to trap there for 2 weeks (Cameron *et al*., unpublished data). In overnight trap sessions, we typically set traps in the evening of the first day, and removed traps on the morning of the final day (overall mean trap effort: 576 ± 58 trap-hours, Fig. S1). We acknowledge that our approach is probably too short to determine whether turtles are absent from a site, and do not consider an absence of detection as an absence of turtles. Instead, our aim was to determine a relative index of turtle population characteristics that could be compared across the catchment.

We used three different turtle trap types: cathedral traps, single-wing fyke traps, and crab pots. We used a variety of traps because each trap type is not equally suited to use in every type of habitat. Cathedral traps are best suited for still water >1.4 m deep, while fyke traps are best suited for still or slow-moving water <1 m deep, and crab pots are best suited for very shallow, slow-moving water <0.2 m deep. At most sites, we used at least 10 traps, and usually mixed trap types (3-4 each), while trapping a site (maximum 25). We set cathedral traps in open, still water deeper than 1 m. We set fyke traps in open water 0.2–0.5 m deep, with the wing of the fyke parallel to the nearest shoreline. We set crab pots in water ~0.2 m deep, sometimes nestled within benthic vegetation. Despite their differences, each trap type is capable of catching at least 10 turtles at a time. When trapping in fast-flowing streams, we always placed traps in or on the edge of deeper pools, where currents slowed as much as possible. All traps were baited with a mix of offal (lamb heart, lamb liver, chicken liver). Traps were checked twice per day and were re-baited once per day.

Upon checking traps, we removed any turtles present and immediately re-set the trap, and re-baited it if necessary. After removing turtles from traps, we checked them for existing carapace marks to determine whether they were recaptures, from our own or previous studies^69^. We recorded the identifications of turtles recaptured within the trapping session and immediately released them. Occasionally, we caught turtles that had been marked by a previous study, potentially up to 40 years earlier e.g., Chessman; Thompson^9,33^. We recorded these turtles’ existing IDs, but considered them novel to our current analyses. We weighed all turtles to the nearest 1 g on a digital scale. We measured straight carapace length and width with forestry callipers. We marked all unidentified turtles individually by notching their marginal scutes^69^. We recorded the sex of each turtle where possible. The tails of adult and subadult *C. expansa* and *E. macquarii* are sexually dimorphic and longer in males. The tails of adult *C. longicollis* are only slightly sexually dimorphic and other diagnostics are not universally reliable, so we did not record sexes of *C. longicollis*^47^. All work was conducted under OEH permit SL101639, DPI permit P09/0070-20, DEWNR Permit M26597-1, DEWLP permit 10007501, DEPI permit RP1225, and Western Sydney University Animal Ethics Committee Animal Research Authority A10455.

### Data Analysis

We analyzed two metrics of turtle population status at each site: catch-per-unit-effort (CPUE) and the proportions of juvenile and large adult turtles of each species. Both metrics were analyzed separately for each species. We used CPUE as a relative index of local abundance to assess the number of turtles present in a site whilst accounting for trapping effort produced by our design. Because we caught at least 25 *E. macquarii* at some sites very rapidly, our trapping effort at those sites was relatively small, whereas our trapping effort was larger at sites where we caught turtles at a slower rate. We compared CPUE of each species across sites using log-linear Analyses of Covariance with Poisson distributions (PROC GLIMMIX) in SAS University Edition (SAS Institute, Cary, NC). We accounted for trapping effort by multiplying the number of traps set by the number of hours they were set, disregarding trap type, and including this value as a covariate. We specified wetland type as an independent variable to test for CPUE differences among wetland types, and approximate river distance on the Murray River (distance in km from the river mouth, hereafter referred to as River Distance) as a covariate to test for geographic differences across the Murray Catchment. In this design, multiple sites sampled from the same tributary (e.g., Loddon River) were assigned the same river distance, because the confluence between the tributary and the Murray River was the same for all. We also included distance from the nearest permanent water body (km), distance from the Murray River, mean annual precipitation (mm) and mean air temperature (°C) to test for environmental and climatic effects on turtle CPUE across the catchment. Distance data were extracted from Google Earth^TM^. Climate data were extracted from Worldclim (Ver. 2, www.worldclim.org)^70^, using location averages to within 30 seconds of latitude/longitude spatial resolution, from 1970–2000. We log-transformed all numerical data in this analysis. Because of the wide variation in trapping effort across sites (Fig. S1), we tested for robustness in our results by running an identical model without sites that had fewer than 100 total trap-hours for any reason, and also without Lake Bonney, because it was an outlier with very high trapping effort. For presentation of the data, we also calculated CPUE by summing the number of turtles caught by all traps at a site and dividing by the number of trap-hours (number of traps multiplied by number of hours traps were set). We calculated CPUE separately for each species, and did not distinguish among sex, body size, or trap type.

We used the numbers of juveniles and large adults, relative to total number caught, as metrics of recent recruitment and population aging, because both drought and fox destruction of nests have been suggested to drive losses of recruitment, which may result in population collapses if adults are not replaced as they die of old age^9,33,48^. Furthermore, size-structure bias toward smaller (younger) or larger (older) turtles may suggest different stressors affecting population dynamics, e.g. low recruitment or population bottlenecks. At any site where we caught at least five turtles of a given species, we counted the numbers of juveniles of each species, using straight-carapace length (SCL), following Chessman^33,47^. The maximum cutoff for juvenile SCL was 220 mm for *C. expansa*, 180 mm for *C. longicollis*, and 190 mm for *E. macquarii*. We also counted the number of large adults of each species using arbitrary cutoffs: 350 mm for *C. expansa*, 230 mm for *C. longicollis*, and 250 mm for *E. macquarii*.

Within each site, we tested for differences in the relative numbers of juveniles and large adults using log-linear Analyses of Covariance with Poisson distributions (PROC GLIMMIX) for each species. We included the total number of turtles caught at each site as a covariate to account for differences in the numbers of turtles caught. We included “sex” (juvenile, large female, large male; or juvenile, large adult in *C. longicollis*) to test for differences in the numbers of each group. As in the CPUE analysis, we included river distance, distance from the Murray River, distance from the nearest permanent water, wetland type, mean annual temperature, and mean annual precipitation to assess geographic and climate relationships in the numbers of juveniles and large adults. We also included the interaction sex*wetland type to test whether numbers of juveniles and large adults differed uniquely within each wetland type. Finally, we included site as a random effect to account for repeated within-site comparisons across sexes. We also calculated percentages of juveniles, large females, and large males (or large adults for *C. longicollis*) at each site in order to present the data clearly. In all analyses, we determined statistical significance at 0.05 type I error level, and we present data as means ± SE.

## Supplementary information

Supplementary Materials

## Data Availability

The datasets generated during and/or analysed during the current study are available from the corresponding author on reasonable request.

## References

[1] Ceballos G (2015). Accelerated modern human–induced species losses: Entering the sixth mass extinction. Science Advances.

[2] Dirzo R (2014). Defaunation in the anthropocene. Science.

[3] Barnosky AD (2011). Has the Earth’s sixth mass extinction already arrived?. Nature.

[4] Lister, B. C. & Garcia, A. Climate-driven declines in arthropod abundance restructure a rainforest food web. *Proceedings of the National Academy of Sciences*, 10.1073/pnas.1722477115 (2018). 10.1073/pnas.1722477115 PMC6217376 30322922

[5] Purvis A, Gittleman JL, Cowlishaw G, Mace GM (2000). Predicting extinction risk in declining species. Proceedings of the Royal Society B, Biological Sciences.

[6] Keith DA (2008). Predicting extinction risks under climate change: coupling stochastic population models with dynamic bioclimatic habitat models. Biology Letters.

[7] Brook BW, Sodhi NS, Bradshaw CJA (2008). Synergies among extinction drivers under global change. Trends Ecol. Evol..

[8] Martin TG (2012). Acting fast helps avoid extinction. Conservation Letters.

[9] Thompson MB (1983). Populations of the Murray River Tortoise, *Emydura* (Chelodina): the effect of egg predation by the Red Fox, *Vulpes vulpes*. Aust. Wildl. Res..

[10] Rhodin, A. G. J. *et al*. *Turtles in Trouble: The World’s 25+ Most Endangered Tortoises and Freshwater Turtles—2011*. (Turtle Conservation Coalition, IUCN/SSC, Lunenburg, MA, USA, 2011).

[11] Colteaux BC, Johnson DM (2017). Commercial harvest and export of snapping turtles (*Chelydra serpentina*) in the United States: trends and the efficacy of size limits at reducing harvest. Journal of Nature Conservation.

[12] Gong S (2017). Disappearance of endangered turtles within China’s nature reserves. Curr. Biol..

[13] Beaudry F, DeMaynadier PG, Hunter ML (2008). Identifying road mortality threat at multiple spatial scales for semi-aquatic turtles. Biol. Conserv..

[14] Langen TA, Gunson KE, Scheiner CA, Boulerice JT (2012). Road mortality in freshwater turtles: identifying causes of spatial patterns to optimize road planning and mitigation. Biodivers. Conserv..

[15] Spencer R-J, Thompson MB (2005). Experimental analysis of the impact of foxes on freshwater turtle populations. Conserv. Biol..

[16] Heinrich GL, Walsh TJ, Jackson DR, Atkinson BK (2012). Boat strikes: a threat to the Suwannee Cooter (*Pseudemys concinna suwanniensis*). Herpetological Conservation and Biology.

[17] Lum LL (2006). Assessment of incidental sea turtle catch in the artisanal gillnet fishery in Trinidad and Tobago, West Indies. Applied Herpetology.

[18] Limpus DJ, Limpus CJ, Hodge WJ (2006). Marine and Freshwater Sciences Environmental Sciences Division. Conservation Technical and Data Report.

[19] Steen DA, Hopkins BC, Van Dyke JU, Hopkins WA (2014). Prevalence of ingested fish hooks in freshwater turtles from five rivers in the southeastern United States. PLoS One.

[20] Steen DA, Robinson OJ (2017). Estimating freshwater turtle mortality rates and population declines following hook ingestion. Conserv. Biol..

[21] Tomillo PS, Saba VS, Piedra R, Paladino FV, Spotila JR (2008). Effects of illegal harvest of eggs on the population decline of leatherback turtles in Las Baulas Marine National Park, Costa Rica. Conserv. Biol..

[22] Spencer RJ, Van Dyke JU, Thompson MB (2016). The ‘Ethological Trap’: Functional and numerical responses of highly efficient invasive predators driving prey extinctions. Ecol. Appl..

[23] Rhymer JM, Simberloff D (1996). Extinction by hybridization and introgression. Annu. Rev. Ecol. Syst..

[24] Georges, A., Spencer, R. -J., Killian, A. & Zhang, X. Assault from all sides: hybridization and introgression threaten the already critically endangered *Myuchelys georgesi* (Chelonia: Chelidae). *Endangered Species Research*, 10.3354/esr00928 (2018).

[25] Spencer RJ, Georges A, Lim D, Welsh M, Reid AM (2014). The risk of inter-specific competition in Australian short-necked turtles. Ecol. Res..

[26] Moloney, B., Britton, S. & Matthews, S. *Bellinger River snapping turtle mortality event 2015: epidemiology report*. (NSW Department of Primary Industries, 2015).

[27] Cann J, Spencer RJ, Welsh M, Georges A (2015). *Myuchelys georgesi* (Cann 1997) – Bellinger River Turtle. Chelonian Conserv. Biol..

[28] Spencer RJ (2018). Profiling a possible rapid extinction event in a long-lived species. Biol. Conserv..

[29] Johnson AJ (2008). Ranavirus infection of free-ranging and captive box turtles and tortoises in the United States. J. Wildl. Dis..

[30] Brown MB (1999). Upper respiratory tract disease in the gopher tortoise is caused by *Mycoplasma agassizii*. Journal of Clinical Microbiology.

[31] Schwanz LE, Janzen FJ (2008). Climate change and temperature-dependent sex determination: can individual plasticity in nesting phenology prevent extreme sex ratios. Physiol. Biochem. Zool..

[32] Gibbons JW, Greene JL, Congdon JD (1983). Drought-related responses of aquatic turtle populations. J. Herpetol..

[33] Chessman BC (2011). Declines of freshwater turtles associated with climatic drying in Australia’s Murray-Darling Basin. Wildl. Res..

[34] Bower DS, Hutchinson M, Georges A (2012). Movement and habitat use of Australia’s largest snake-necked turtle: implications for water management. J. Zool. (Lond.).

[35] Bower DS, Death CE, Georges A (2012). Ecological and physiological impacts of salinisation on freshwater turtles of the lower Murray River. Wildl. Res..

[36] Bower DS (2016). Salinity tolerances of two Australian freshwater turtles, *Chelodina expansa* and *Emydura macquarii* (Testudinata: Chelidae). *Conservation*. Physiology.

[37] Van Dyke JU, Hopkins WA, Jackson BP (2013). Influence of relative trophic position and carbon source on selenium bioaccumulation in turtles from a coal fly-ash spill site. Environ. Pollut..

[38] Bergeron CM, Husak JF, Unrine JM, Romanek CS, Hopkins WA (2007). Influence of feeding ecology on blood mercury concentrations in four species of turtles. Environ. Toxicol. Chem..

[39] Hopkins BC, Willson JD, Hopkins WA (2013). Mercury exposure is associated with negative effects on turtle reproduction. Environ. Sci. Technol..

[40] Van Dyke JU, Beck ML, Jackson BP, Hopkins BC (2013). Interspecific differences in egg production affect egg trace element concentrations after a coal fly ash spill. Environ. Sci. Technol..

[41] Ferronato BO, Roe JH, Georges A (2014). Reptile bycatch in a pest-exclusion fence established for wildlife reintroductions. Journal for Nature Conservation.

[42] Ferronato BO, Roe JH, Georges A (2016). Urban hazards: spatial ecology and survivorship of a turtle in an expanding suburban environment. Urban Ecosyst..

[43] Brooks RJ, Brown GP, Galbraith DA (1991). Effects of a sudden increase in natural mortality of adults on a population of the common snapping turtle (Chelydra serpentina). Can. J. Zool..

[44] Spencer R-J, Van Dyke JU, Thompson MB (2017). Critically evaluating best management practices for preventing freshwater turtle extinctions. Conserv. Biol..

[45] Keevil MG, Brooks RJ, Litzgus J (2018). Post‐catastrophe patterns of abundance and survival reveal no evidence of population recovery in a long‐lived animal. Ecosphere.

[46] Robley A (2016). The effectiveness of short-term fox control in protecting a seasonally vulnerable species, the Eastern Long-necked Turtle (*Chelodina longicollis*). Ecol. Manag. Restor..

[47] Chessman, B. C. *Ecological studies of freshwater turtles in south-eastern Australia*., Monash University (1978).

[48] Thompson, M. B. In *Herpetology in* Australia (eds D. Lunney & D. Ayes) 219–224 (Surrey Beatty and Sons, 1993).

[49] Van Dyke, J. U., Ferronato, B. & Spencer, R. J. Current conservation status of Australian freshwater turtles. *Aust. J. Zool*. **66**, 1–3 (2018).

[50] Chessman BC (1988). Habitat preferences of fresh-water turtles in the Murray Valley, Victoria and New South Wales. Wildl. Res..

[51] Ocock JF (2017). Identifying critical habitat for Australian freshwater turtles in a large regulated floodplain: implications for environmental water management. Environ. Manag..

[52] Roe JH, Georges A (2008). Terrestrial activity, movements and spatial ecology of an Australian freshwater turtle, *Chelodina longicollis*, in a temporally dynamic wetland system. Austral. Ecol..

[53] Roe JH, Georges A (2008). Maintenance of variable responses for coping with wetland drying in freshwater turtles. Ecology.

[54] Spencer R-J, Janzen FJ, Thompson MB (2006). Counterintuitive density-dependent growth in a long-lived vertebrate after removal of nest predators. Ecology.

[55] Kingsford RT (2000). Ecological impacts of dams, water diversions and river management on floodplain wetlands in Australia. Austral. Ecol..

[56] Koehn JD (2004). Carp (*Cyprinus carpio*) as a powerful invader in Australian waterways. Freshw. Biol..

[57] Petrov, K., Lewis, J., Malkiewicz, N., Van Dyke, J. U. & Spencer, R. J. Food abundance and diet variation in freshwater turtles from the mid-Murray River, Australia. *Aust. J. Zool*. **66**, 67–76 (2018).

[58] Skinner D, Oliver R, Aldridge K, Brookes J (2014). Extreme water level decline effects sediment distribution and composition in Lake Alexandrina, South Australia. Limnology.

[59] Morrongiello JR, Crook DA, King AJ, Ramsey DSL, Brown P (2011). Impacts of drought and predicted effects of climate change on fish growth in temperate Australian lakes. Global Change Biology.

[60] Kingsley, T. -A. In *ABC Central Victoria*, http://www.abc.net.au/local/stories/2007/05/01/1910984.htm (2007).

[61] Humphries, P. Loddon-Campaspe drought response options – environmental flow management. (North Central Catchment Management Authority, Bendigo, VIC, Loddon River Environmental Flows Panel, 2006).

[62] Spencer R-J (2002). Growth patterns of two widely distributed freshwater turtles and a comparison of common methods used to estimate age. Aust. J. Zool..

[63] Lovich, J. E., Ennen, J. R., Agha, M. & Gibbons, J. W. Where have all the turtles gone, and why does it matter? *Bioscience*, 10.1093/biosci/biy1095 (2018).

[64] Stokeld D, Hamer AJ, van der Ree R, Pettigrove V, Gillespie G (2014). Factors influencing occurrence of a freshwater turtle in an urban landscape: a resilient species?. Wildl. Res..

[65] Jergensen AM, Miller DAW, Neuman-Lee LA, Warner DA, Janzen FJ (2014). Swimming against the tide: resilience of a riverine turtle to recurrent extreme environmental events. Biology Letters.

[66] Mitchell JC (1988). Population ecology and life histories of the freshwater turtles *Chrysemys picta* and *Sternotherus odoratus* in an urban lake. Herpetol. Monogr..

[67] Lamb T, Bickham JW, Lyne TB, Gibbons JW (1995). The slider turtle as an environmental sentinel: multiple tissue assays using flow cytometric analysis. Ecotoxicology.

[68] Hopkins BC, Hepner MJ, Hopkins WA (2013). Nondestructive techniques for biomonitoring of spatial, temporal, and demographic patterns of mercury bioaccumulation and maternal transfer in turtles. Environ. Pollut..

[69] Cagle FR (1939). A system of marking turtles for future identification. Copeia.

[70] Hijmans RJ, Cameron SE, Parra JL, Jones PG, Jarvis A (2005). Very high resolution interpolated climate surfaces for global land areas. International Journal of Climatology.

