# Supplementary material for: Conservation implications of turtle declines in Australia’s Murray River system
Source: Sci Rep. 2019 Feb 13;9:1998. doi: 10.1038/s41598-019-39096-3 (PMC6374471; doi:10.1038/s41598-019-39096-3)
Supplement: Supplementary file 1 — Supplementary Materials [file 41598_2019_39096_MOESM1_ESM.pdf]

Supplemental Materials for:

Conservation implications of turtle declines in Australia's Murray River system

J.U. Van Dyke[1,2], *R-J Spencer[1], M.B Thompson[3], B. Chessman[4], K. Howard[1] and A. Georges[5].

1. School of Science and Health, Hawkesbury Institute for the Environment, Western Sydney University, Locked Bag 1797, Penrith NSW, 2751 Australia
2. School of Environmental Sciences, Institute for Land, Water, and Society, Charles Sturt University, Albury Wodonga Campus Albury NSW 2640 Australia
3. School of Life and Environmental Sciences, Heydon-Laurence Building (A08), University of Sydney, NSW 2006 Australia
4. Centre for Ecosystem Science, University of New South Wales, Sydney NSW 2052, Australia
5. Institute for Applied Ecology, University of Canberra, ACT 2601 Australia

Email: R.Spencer@westernsydney.edu.au

Running Head: Decline of Australian River Turtles

Keywords: *Emydura macquarii, Chelodina longicollis, Chelodina expansa, invasive species, habitat fragmentation, recruitment, attrition*

Table S1. Statistical results of log-linear ANCOVA analyses on the CPUE of each turtle species in the southern Murray River catchment. Factors that were statistically significant in each model are in bold font. This analysis does not include sites with low trapping effort (<100 trap-hours) or exceptionally high trapping effort (Lake Bonney).

Species	Effect	F	Num df	Den df	P
C. expansa	Distance to Murray River	0.74	1	30	0.400
	Distance to Permanent	0.01	1	30	0.990
	Mean Precipitation	0.65	1	30	0.425
	Mean Temperature	1.13	1	30	0.297
	River Kilometre	3.24	1	30	0.082
	Trapping Effort	0.46	1	30	0.502
	Wetland Type	0.81	5	30	0.549
C. longicollis	Distance to Murray River	0.20	1	30	0.661
	Distance to Permanent	0.35	1	30	0.558
	Mean Precipitation	0.01	1	30	0.905
	Mean Temperature	0.26	1	30	0.611
	River Kilometre	1.30	1	30	0.264
	Trapping Effort	0.05	1	30	0.831
	**Wetland Type**	**4.75**	**5**	**30**	**0.003**
E. macquarii	Distance to Murray River	0.13	1	30	0.724
	Distance to Permanent	0.01	1	30	0.916
	Mean Precipitation	0.67	1	30	0.421
	Mean Temperature	0.67	1	30	0.419
	**River Kilometre**	**5.11**	**1**	**30**	**0.031**
	Trapping Effort	0.70	1	30	0.411
	**Wetland Type**	**3.65**	**5**	**30**	**0.011**

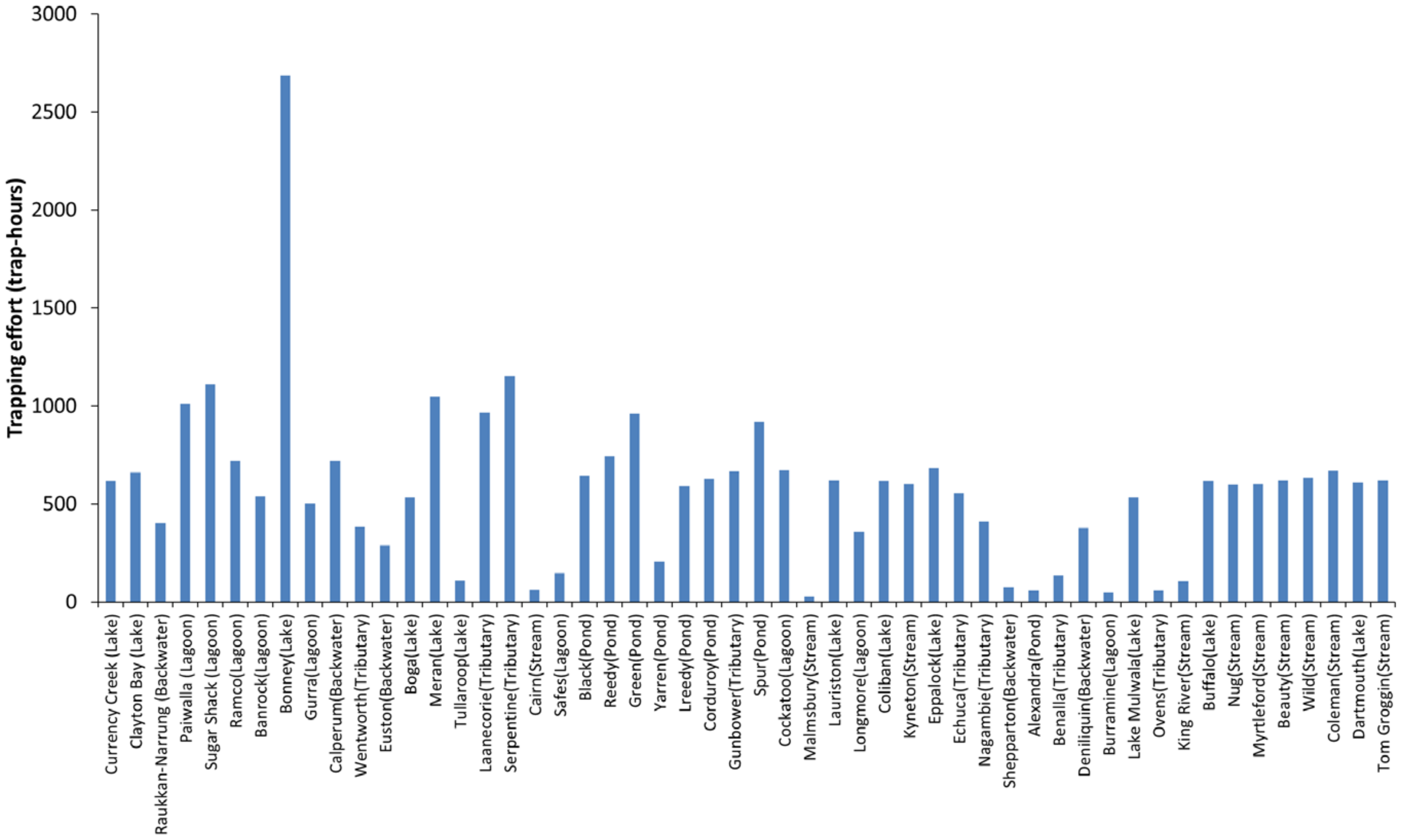

Figure S1. Trapping effort (number of traps multiplied by number of hours set) at each site in our study.

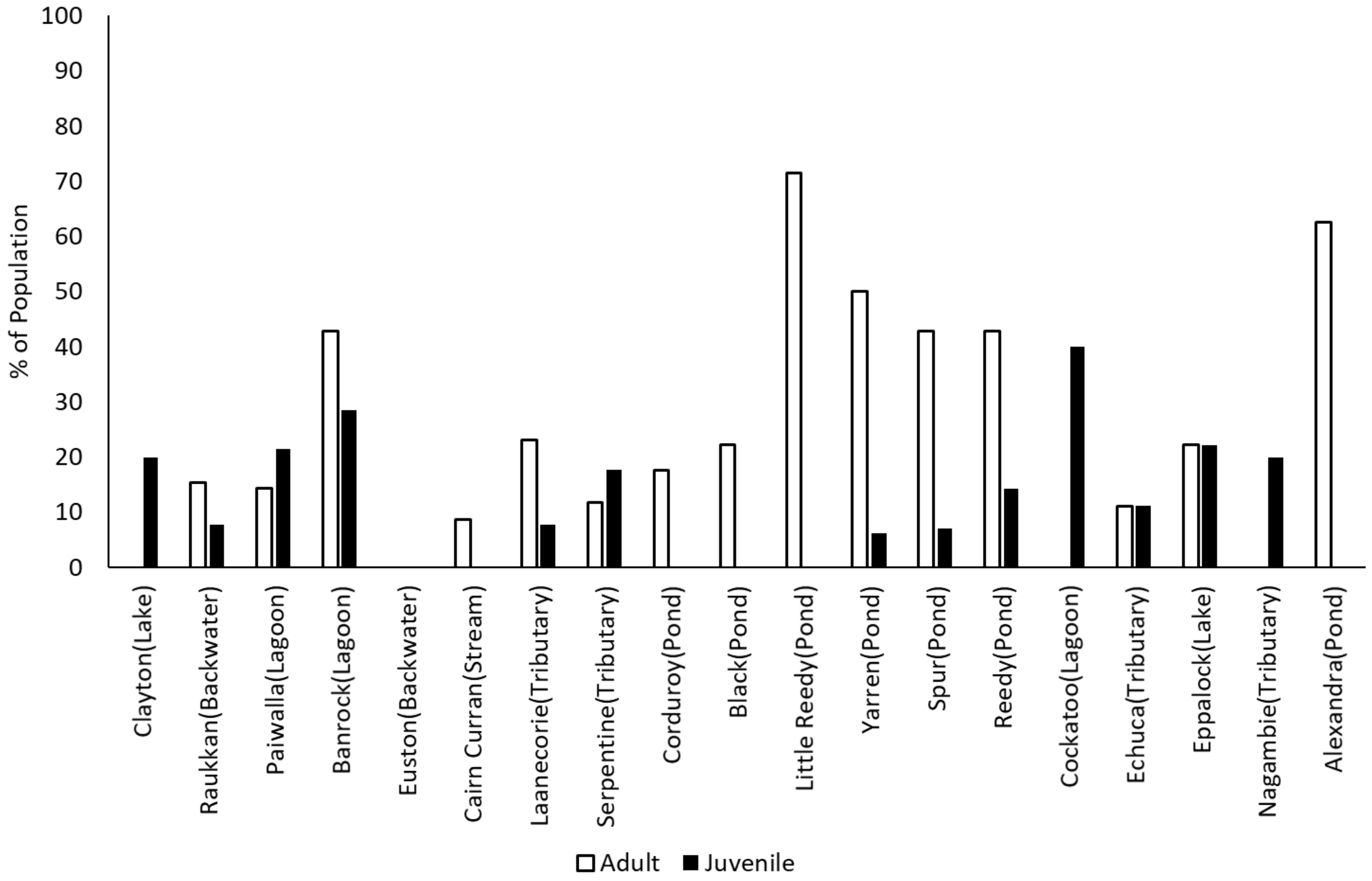

Figure S2. *Chelodina longicollis* population percentages of large adults and juveniles at each site where we caught sufficient numbers for our demographic analysis. Sites are ordered left-right from downstream-upstream. Mean percentages do not sum to 100% because we did not include the percentages of intermediate-sized turtles in these analyses.

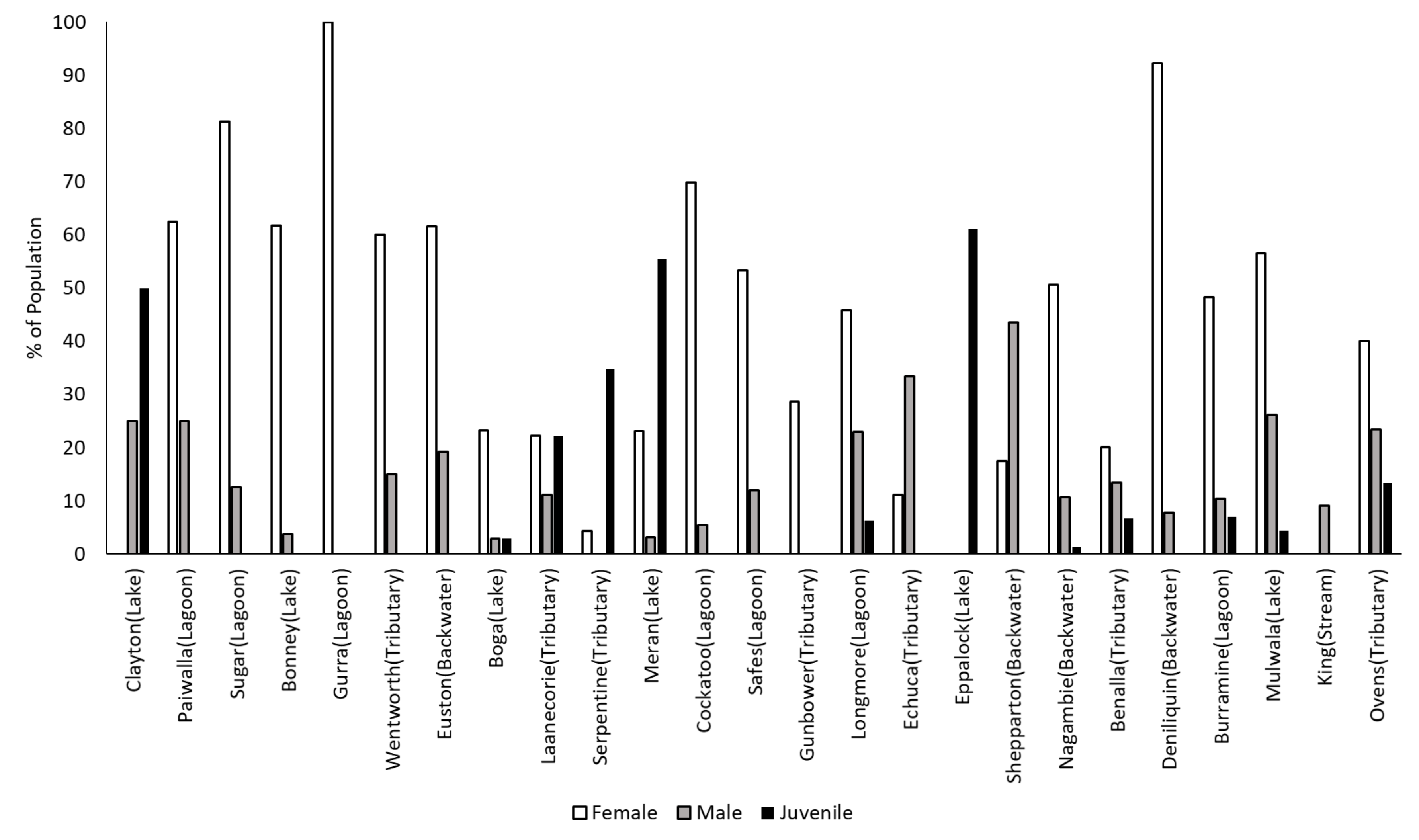

Figure S3. *Emydura macquarii* population percentages of large females, large males, and juveniles at each site where we caught sufficient numbers for our demographic analysis. Sites are ordered left-right from downstream-upstream. Mean percentages do not sum to 100% because we did not include the percentages of intermediate-sized turtles in these analyses.
